# Magnetic resonance imaging findings in children with Parry-Romberg syndrome and en coup de sabre

**DOI:** 10.1186/s12969-021-00512-6

**Published:** 2021-03-23

**Authors:** Harry Knights, Elizabeth Minas, Faraan Khan, Lindsay Shaw, Muthana Al Obaidi, Kshitij Mankad, Despina Eleftheriou

**Affiliations:** 1 Department of Paediatric Rheumatology, University College London Institute of Child Health and Great Ormond Street Hospital for Children NHS Foundation Trust, London, UK; 2 Imperial College School of Medicine, Imperial College London, London, UK; 3 Department of Radiology, Charing Cross Hospital, London, UK; 4 Department of Radiology, St George’s University Hospitals NHS Foundation Trust, London, UK; 5 Department of Paediatric Dermatology, University College London Institute of Child Health and Great Ormond Street Hospital for Children NHS Foundation Trust, London, UK; 6 Department of Radiology, Great Ormond Street Hospital for Children NHS Foundation Trust, London, UK; 7 Centre for Adolescent Rheumatology Versus Arthritis, University College London, London, UK

**Keywords:** Parry-Romberg syndrome, En coup de sabre, Linear scleroderma, Magnetic resonance imaging, Epilepsy

## Abstract

**Background:**

The aim of this study was to: (i) describe the abnormalities seen on brain imaging in a group of children with en coup de sabre (EDCS) with/without Parry-Romberg syndrome (PRS); and (ii) identify clinical predictors of brain imaging abnormalities.

**Methods:**

This was a single centre (Great Ormond Street Hospital, London) retrospective case series of patients with ECDS/PRS seen from 2000 to 2018. We identified patients with cutaneous manifestations consistent with the clinical descriptions of ECDS/PRS. Presenting clinical, laboratory, and radiological brain findings are described. Results are expressed as medians and ranges or frequencies and percentages. Fisher’s exact test was used to identify clinical associations with magnetic resonance imaging (MRI) abnormalities.

**Results:**

Fourteen patients were studied: 6 males and 8 females; median age 14 years (range 3–20). We observed neuroimaging abnormalities in 2/6 ECDS and 5/8 ECDS/PRS patients. White matter signal abnormality, dystrophic calcification, leptomeningeal enhancement, and sulcal crowding were the typical findings on brain imaging. A total of 50% of patients had no MRI abnormality despite some of these patients having neurological symptoms. The presence of seizures was significantly associated with ipsilateral enhanced white matter signalling on MRI (*p* < 0.05).

**Conclusions:**

In summary, we observed several distinct radiographic patterns associated with ECDS/PRS. Seizure disorder was strongly associated with the presence of ipsilateral enhanced white matter signalling. Improved neuroimaging techniques that combine morphological with functional imaging may improve the detection rate of brain involvement in children with ECDS/PRS in the future.

**Supplementary Information:**

The online version contains supplementary material available at 10.1186/s12969-021-00512-6.

## Background

En coup de sabre (ECDS, “from a blow of the sword”) is a type of linear scleroderma which begins as a line of cutaneous inflammation and develops into a line of fibrosis (as is the usual pattern for linear scleroderma anywhere on the body) [1]. Although ECDS tends to start on the scalp, this line may extend downwards to involve inferior areas such as the forehead, brow ridge, eyelid and its appendages, cheek, dental ridge, and chin [2]. Parry-Romberg syndrome (PRS) is a diffuse hemifacial atrophy [3]. It can be subtle at first but progresses over time to involve the skin, soft tissues, and underlying bone [3]. It almost always affects a larger area than ECDS, and affected skin has absent or minimal fibrosis which can be appreciated on examination and demonstrated on skin biopsy [3]. PRS also tends to become more prominent with age as the affected tissues fail to grow as well as the unaffected side [3]. ECDS and PRS may occur alone, but patients with ECDS may subsequently develop PRS as well [4]. Changes of cutaneous ECDS or PRS may be subtle on imaging studies, but 20% of these patients will have intracranial manifestations that may not correspond to the severity of soft-tissue involvement or neurological symptoms [5]. The recently published Single Hub Access Point for Paediatric Rheumatology (SHARE) consensus-based recommendations for the management of juvenile localised scleroderma recognised this lack of correlation between the severity of cutaneous disease and central nervous system (CNS) involvement and recommended that all patients with juvenile linear scleroderma such as ECDS and PRS involving the face and head, with or without signs of neurological involvement, should have magnetic resonance imaging (MRI) of the head at the time of diagnosis [6]. These recommendations did not, however, provide any guidance on the specific imaging findings associated with neurological symptoms in ECDS/PRS due to a lack of relevant data: most imaging studies to date have been reported in adults, with only single case reports or small cases series in childhood disease [7–9]. The aim of this study was therefore to: (i) describe the CNS imaging abnormalities seen in a group of children with ECDS with/without PRS; and (ii) identify clinical predictors for the development of these MRI abnormalities.

## Methods

### Patients

This was a retrospective case notes review collating anonymised clinical information obtained as part of routine clinical care. Diagnosis was made in patients with cutaneous symptoms consistent with the clinical descriptions of ECDS/PRS: lateral forehead scleroderma with/without hemifacial atrophy. We used electronic institutional clinical records to identify all patients with a diagnosis of ECDS or PRS seen at Great Ormond Street Hospital (GOSH), London, between January 2000 and December 2018. The demographic, clinical, and laboratory characteristics recorded were as follows: sex; age; age at diagnosis; ethnicity (established via the information provided by patients/parents upon registering with the hospital); neurological manifestations; other system involvement; presence of serum autoantibodies; cerebrospinal fluid (CSF) examination; and electroencephalogram (EEG). Therapies used were also recorded.

### Neuroimaging acquisition and review

All patients had undergone basic magnetic resonance (MR) imaging including axial T2-weighted imaging, coronal fluid-attenuated inversion recovery (FLAIR) imaging, sagittal T1-weighted imaging and axial diffusion weighted imaging (DWI). All patients with MR imaging received gadolinium contrast on at least one follow up scan. Computed tomography (CT) or MR susceptibility weighted imaging (SWI) was available for the majority of patients to assess dense mineralisation.

MR imaging was performed at diagnosis in 13 patients, and 7 years after diagnosis in one patient without neurological manifestations. Follow up imaging was performed to assess for resolution or progression of imaging abnormalities at 6–12 month intervals and when there was acute neurological symptomatology.

Two neuroradiologists (KM and FK) blinded to the clinical status of the patients reviewed the imaging for the following specific abnormalities: white matter hyperintensities on T2-weighted imaging/FLAIR; calcification on CT/SWI; cerebral atrophy on T1-weighted imaging; and leptomeningeal enhancement post contrast administration. Any additional findings that were relevant were also recorded. Sequential imaging, where available, was also reviewed and assessed for white matter signal and calcification changes (stability, progression, improvement, and mixed responses).

### Statistical analysis

Descriptive statistics were used: continuous variables were summarised as median and range; categorical variables were presented as frequencies and percentages. Parameters between groups were compared using the Fisher’s exact test. A two-sided *p* value < 0.05 was considered statistically significant. Statistical analysis was done with IBM SPSS statistics version 21.

## Results

### Demographics

A total of 14 patients were included in the study: 6 (43%) males and 8 (57%) females; median age 14 years (range 3–20 years). A total of 9/14 (64%) were white British, one was Pakistani, one was Indian, one was white Irish, and 2/14 (18%) were of unknown ethnic origin. Median age at the time of initial diagnosis was 7 years (range 5 months–14 years), with a median time from disease onset of 5 years (range 1–12 years). Six patients had ECDS alone; 8 patients had ECDS with PRS; and none had PRS alone.

### Additional clinical features

A summary of clinical features additional to the cutaneous manifestations, related or unrelated to ECDS/PRS, is provided in Table 1.

**Table 1:** Additional clinical manifestations in patients with Parry-Romberg syndrome (PRS) and en coup de sabre (ECDS)

System	Manifestation	Patients (***n*** = 14)
**Any**	Any	12
**Neurological**	Any	9
	Seizure	6
	Headache	4
	Cranial nerve dysfunction	2
	TIA	1
	Myoclonus	1
	Hyperaesthesia	1
	Gait disturbance	1
	Visual loss	1
**Psychiatric**	Any	4
	Anxiety	2
	Depression	1
	OCD	1
	Visual hallucinations	1
**Cardiovascular**	Any	2
	Hypertension	1
	Bicuspid aortic valve	1
**Articular**	TMJ dysfunction	1
**Muscular**	Myositis	1
**Gastro-intestinal**	Gastro-oesophageal reflux	1
**Vascular**	Raynaud’s phenomenon	0
**Respiratory**	Any	0

*TIA* Transient ischaemic attack, *OCD* Obsessive compulsive disorder, *TMJ* temporomandibular joint

### Laboratory and other investigations

Antinuclear antibodies were positive in 7/12 (58% of patients tested; range 1:160 to 1:2560). Anti-smooth muscle antibodies were positive in 1/5 (20%); anti-rheumatoid factor antibodies were positive in 1/6 (17%); and anti-glutamic acid decarboxylase (GAD) antibodies were positive in 1/1. All other autoantibodies were negative in all patients tested (Supplementary Table S1).

CSF examination was performed in 3 patients: 2 were found to have normal levels of cells and protein; one patient was found to have positive CSF oligoclonal bands and low 5-methyltetrahydrofolate neurotransmitter (51 nmol/l, normal range 72–172).

EEG was performed in 5/6 patients with seizures. Four/5 (80% of EEGs) had documented abnormalities: all displayed intermittent slowing of the left posterior region; 3/4 (75%) displayed epileptiform activity, although this was only associated with a clinical event in one patient.

### Imaging abnormalities and neurological manifestations

**Fig. 1:**
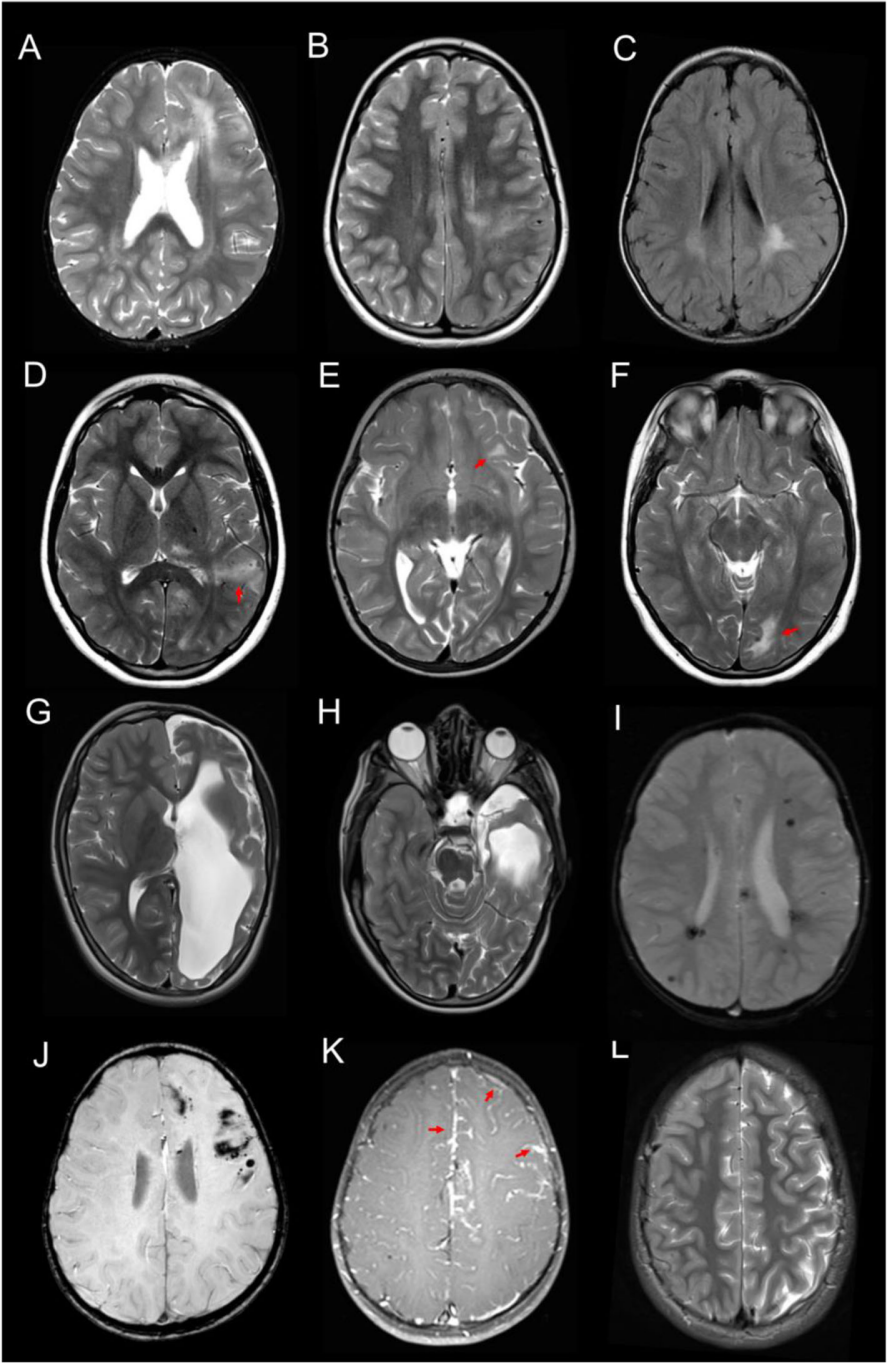
Magnetic resonance imaging (MRI) abnormalities in patients with Parry-Romberg syndrome and en coup de sabre. **a**-**f** T2-weighted and FLAIR MRI images showing enhanced white matter signalling. **g**-**h** T2-weighted MRI image showing porencephaly. **i** T2-weighted gradient echo MRI image showing multiple cavernomata. **j** T2-weighted gradient echo MRI image showing dystrophic calcification. **k** T1-weighted gadolinium enhanced MRI image showing leptomeningeal enhancement along the medial and lateral surfaces of the left frontal lobe. **l** T2-weighted MRI image showing sulcal crowding on the right

MRI abnormalities were noted in 2/6 ECDS and 5/8 ECDS/PRS patients (Table 2). A combined analysis of the imaging findings is presented (Tables 3 and 4; Fig. 1).

**Table 2:** Magnetic resonance imaging (MRI) abnormalities and progression in individual patients with Parry-Romberg (PRS) and en coup de sabre (ECDS)

	Demographics			Additional clinical features			MRI findings					
ID	Skin	Sex	Age at diagnosis/ first scan (years)	Neurological	Psychiatric	Other	White matter signal	White matter volume	Calcification	Enhancement	Other findings	Follow up imaging
**1**	ECDS	M	6/6	Headaches	Visual hallucinations	Gastro-oesophageal reflux	Normal	Preserved	Absent	Absent		Unchanged at 1 year
**2**	ECDS	F	2/2	Seizures; Cranial nerve dysfunction	Obsessive compulsive disorder		Abnormal; Mixed response	Preserved with sulcal crowding	Present; Progressive	Present; Leptomeningeal; Increases		3 years
**3**	ECDS	F	12/9	Seizures; Headaches; Transient ischaemic attack; Hyperaesthesia	Depression; Anxiety		Abnormal; Regressive	Preserved	Present; Progressive	Present; Leptomeningeal; Increases		6 years
**4**	ECDS	M	3/3	Headaches			Normal	Preserved	Absent	Absent		Unchanged at 5 years
**5**	ECDS	M	0.4/0.9				Normal	Preserved	Absent	Absent		No follow up imaging
**6**	ECDS	F	8/9	Seizures		Myositis	Normal	Preserved	Absent	Absent		Unchanged at 1 year
**7**	ECDS/PRS	F	7/3	Seizures; Cranial nerve dysfunction; Gait disturbance; Myoclonus			Abnormal; Regressive	Preserved	Absent	Absent	Left orbital myositis	6 years
**8**	ECDS/PRS	M	2/2	Seizures			Abnormal; Regressive	Preserved	Present; In cavernomata; Stable	Present; In cavernomata; Stable		2 years
**9**	ECDS/PRS	M	6/6	Headaches			Abnormal; Progressive	Preserved with sulcal crowding	Present; Progressive	Present; Leptomeningeal; Stable		11 years
**10**	ECDS/PRS	F	1/0.3	Seizures		Hypertension	Abnormal; Progressive	Progressive atrophy	Present; Stable	Present; Subtle; Leptomeningeal; Unable to assess longitudinally	Porencephaly; Left ocular atrophy	11 years
**11**	ECDS/PRS	F	9/17		Anxiety		Normal	Preserved	Absent	NA		2 years
**12**	ECDS/PRS	F	14/14				Normal	Preserved with sulcal crowding	NA	NA		No follow up imaging
**13**	ECDS/PRS	F	11/7				Normal	Preserved	Absent	NA		5 years
**14**	ECDS/PRS	M	13/14			Temporomandibular joint dysfunction; Bicuspid aortic valve	Normal	Preserved	Absent	NA		4 years

**Table 3:** Predictors of abnormal brain magnetic resonance imaging (MRI) in patients with Parry-Romberg (PRS) and en coup de sabre (ECDS)

				Normal MRI	Abnormal MRI	***p***
**Patient demographics**		**Disease**	**ECDS**	4	2	0.59
			**ECDS/PRS**	3	5	
		**Sex**	**M**	4	2	0.59
			**F**	3	5	
**Additional clinical features**	**Any**	**Any**	**Yes**	5	7	0.46
			**No**	2	0	
	**Neurological**	**Any**	**Yes**	3	6	0.27
			**No**	4	1	
		**Seizures**	**Yes**	1	5	0.1
			**No**	6	2	
		**Headaches**	**Yes**	2	2	1
			**No**	5	5	
		**Cranial nerve dysfunction**	**Yes**	0	2	0.46
			**No**	7	5	
		**Transient ischaemic attack**	**Yes**	0	1	1
			**No**	7	6	
		**Myoclonus**	**Yes**	0	1	1
			**No**	7	6	
		**Hyperaesthesia**	**Yes**	1	0	1
			**No**	6	7	
		**Gait disturbance**	**Yes**	0	1	1
			**No**	7	6	
	**Psychiatric disorder(s)**	**Any**	**Yes**	2	2	1
			**No**	5	5	
		**Anxiety**	**Yes**	1	1	1
			**No**	6	6	
		**Depression**	**Yes**	0	1	1
			**No**	7	6	
		**OCD**	**Yes**	0	1	1
			**No**	7	6	
		**Visual hallucinations**	**Yes**	1	0	1
			**No**	6	7	
	**Cardiovascular**	**Any**	**Yes**	1	1	1
			**No**	6	6	
		**Hypertension**	**Yes**	0	1	1
			**No**	7	6	
		**Bicuspid aortic valve**	**Yes**	1	0	1
			**No**	6	7	
	**Articular**	**TMJ dysfunction**	**Yes**	1	0	1
			**No**	6	7	
	**Muscular**	**Myositis**	**Yes**	1	0	1
			**No**	6	7	
**Laboratory and other investigations**	**Autoantibodies**	**ANA**	**Yes**	3	4	1
			**No**	3	2	
		**Anti-smooth muscle**	**Yes**	0	1	0.4
			**No**	3	1	
		**RF**	**Yes**	0	1	0.33
			**No**	4	1	
		**ENA**	**Yes**	0	0	1
			**No**	1	5	
		**dsDNA**	**Yes**	0	0	1
			**No**	3	4	
	**Cerebrospinal fluid**	**Inflammatory infiltrate**	**Yes**	0	0	1
			**No**	0	3	
	**Neurophysiology**	**EEG abnormality**	**Yes**	1	3	1
			**No**	0	1	

*OCD* obsessive compulsive disorder, *TMJ* temporomandibular joint, *ANA* antinuclear antibody, *RF* rheumatoid factor, *ENA* extractable nuclear antigens, *dsDNA* double stranded DNA, *EEG* electroencephalography

**Table 4:** Predictors of specific brain magnetic resonance imaging (MRI) abnormalities in patients with Parry-Romberg syndrome (PRS) and en coup de sabre (ECDS)

				Demographics															Neurological disorders												Psychiatric disorders						Laboratory		
				Age			Age at diagnosis			Ethnicity			Disease type			Sex			Any			Seizures			Headaches			Cranial nerve dysfunction			Any			Anxiety			ANA		
				< 14	> 14	***p***	< 7	> 7	***p***	White British	Other	***p***	ECDS	ECDS/PRS	***p***	M	F	***p***	Yes	No	***p***	Yes	No	***p***	Yes	No	***p***	Yes	No	***p***	Yes	No	***p***	Yes	No	***p***	Yes	No	***p***
**Specific MRI changes**	**White matter high signal intensity**	***n*** **= 6**	**Yes**	4	2	0.59	4	2	0.59	3	3	0.18	2	4	0.63	2	4	0.63	6	0	**0.031**	5	1	**0.026**	2	4	1	2	4	0.16	2	4	1	1	5	1	3	2	1
			**No**	3	5		3	5		6	0		4	4		4	4		3	5		1	7		2	6		0	8		2	6		1	7		4	3	
	**Dystrophic calcification**	***n*** **= 5**	**Yes**	3	2	1	4	1	0.27	3	2	0.52	2	3	1	2	3	1	5	0	0.1	4	1	0.1	2	3	1	1	4	1	2	3	1	1	4	1	2	2	1
			**No**	4	4		3	5		6	1		4	4		4	4		4	4		2	6		2	6		1	7		2	6		1	7		4	3	
	**Contrast enhancement**	***n*** **= 5**	**Yes**	3	2	1	4	1	1	3	2	1	2	3	0.52	2	3	1	5	0	1	4	1	0.52	2	3	1	1	4	1	2	3	1	1	4	1	2	2	1
			**No**	4	1		3	2		4	1		4	1		3	2		4	1		2	3		2	3		1	4		1	4		0	5		2	2	
	**Leptomeningeal enhancement**	***n*** **= 4**	**Yes**	2	2	0.5	3	1	1	2	2	0.5	2	2	1	1	3	0.52	4	0	1	3	1	0.57	2	2	1	1	3	1	2	2	0.5	1	3	0.4	2	2	1
			**No**	5	1		4	2		5	1		4	2		4	2		5	1		3	3		2	4		1	5		1	5		0	6		2	2	
	**Sulcal crowding**	***n*** **= 3**	**Yes**	1	2	1	2	1	1	1	1	0.45	1	2	1	1	2	1	2	1	1	1	2	1	1	2	1	1	2	0.4	1	2	1	0	3	1	2	1	1
			**No**	6	5		5	6		8	2		5	6		5	6		7	4		5	6		3	8		1	10		3	8		2	9		5	4	
	**Cavernomata**	***n*** **= 1**	**Yes**	1	0	1	1	0	1	1	0	1	0	1	1	1	0	0.43	1	0	1	1	0	0.43	0	1	1	0	1	1	0	1	1	0	1	1	0	0	1
			**No**	6	7		6	7		8	3		6	7		5	8		8	5		5	8		4	9		2	11		4	9		2	11		7	5	
	**Porencephaly**	***n*** **= 1**	**Yes**	1	7	1	1	0	1	1	0	1	0	1	1	0	1	1	1	0	1	1	0	0.43	0	1	1	0	1	1	0	1	1	0	1	1	0	1	0.42
			**No**	6	0		6	7		8	3		6	7		6	7		8	5		5	8		4	9		2	11		4	9		2	11		7	4	

Groups were compared using Fisher’s exact test; *p* < 0.05 was considered significant; significant results are highlighted in bold. *M* Male, *F* Female, *ANA* Anti-nuclear antibody. Contrast enhancement and dystrophic calcification could not be assessed in four and one patients respectively

Six/14 patients demonstrated white matter signal abnormality which regressed over time in 3 cases, progressed in 2, and showed a mixed response in one where the signal improved in parts and progressed in other parts. One of these patients was found to have porencephalic dilatation of the left lateral ventricle at baseline along with ipsilateral Wallerian degeneration. These abnormalities were suggestive of an early life (potentially antenatal) insult, and developed into progressive hemispheric atrophy over time.

An unusual pattern of sulcal crowding subjacent to the morphea was shown in 3 of the 8 PRS patients. Whilst this is not typical of cortical malformation per se, the possibility of dysgyria cannot be excluded.

The presence of dystrophic calcification could be assessed in 6/7 patients with imaging abnormalities and was present in 5. Calcification was noted to be in parenchymal and/or leptomeningeal locations. Progressive calcification was demonstrable in 3 of these 5 cases.

Contrast enhancement was also assessed in 6/7 patients with imaging abnormalities and was found in 5 patients, all of whom also had dystrophic calcification. Contrast enhancement was noted to be in leptomeningeal locations except in one case where it was present surrounding the associated finding of multiple cavernomata.

All patients with neuroradiological findings had abnormalities ipsilateral to the scalp morphea, apart from the patient with cavernomata in whom the findings were bilateral.

Seven/14 (50%) displayed no imaging abnormality, despite some having neurological symptoms including headaches (*n* = 2) and seizures (*n* = 1); and psychiatric symptoms (*n* = 2).

Of the 7/14 (50%) with abnormal imaging, neurological symptoms included: seizures (*n* = 5); headaches (*n* = 2); cranial nerve palsy (*n* = 2); transient ischaemic attack (*n* = 1); myoclonus (*n* = 1); hyperaesthesia (*n* = 1); gait disturbance (*n* = 1); and psychiatric disorder (*n* = 2).

The only MRI abnormality in the 5 patients without neurological symptomatology was sulcal crowding in one patient.

Out of the 10 patients with neurological and/or psychiatric manifestations, 6 had abnormal imaging, all of which had follow up scans. There was no apparent correlation between changes to white matter signal and seizures over time.

### Demographic, clinical and laboratory predictors of MRI brain abnormalities in ECDS/PRS

There were no associations identified between an abnormal MR brain scan and any demographic, clinical, or laboratory parameters (Table 3). The presence of neurological disease (*p* = 0.031), and specifically seizures (*p* = 0.026), were associated with the presence of ipsilateral enhanced white matter signalling on MR brain imaging (Table 4).

### Treatment

The treatment received included: corticosteroids (30 mg/kg/day of IV methylprednisolone given as 3 pulsed doses, followed by 2 mg/kg/day oral prednisolone weaned over 3–6 months) in all patients; methotrexate (15 mg/m^2^/week subcutaneous) in all patients except one; and mycophenolate mofetil (600 mg/m^2^ twice a day oral) in 5 patients. The 5 patients with seizures received a variety of different antiepileptic medications.

## Discussion

In this retrospective case series of 14 patients with ECDS/PRS we have characterised the MR brain abnormalities in ECDS/PRS with onset in childhood. A total of 50% of patients displayed MR brain abnormalities with the typical findings being white matter signal abnormality (43%), dystrophic calcification (36%), leptomeningeal enhancement (29%), and sulcal crowding (21%). We have shown that the development of seizures is significantly associated with ipsilateral enhanced white matter signalling on MRI brain (*p* < 0.05). Prospective studies are now needed to establish the relationship between the detection of MR brain abnormalities and the clinical course of these patients to determine whether escalation of anti-inflammatory treatment leads to prevention of neurological deterioration, such as the development of seizures.

A comparison between our data and those from previous small case series on paediatric ECDS/PRS is present in Table 5 [9–11]. Our observations are consistent with previous studies in adults showing that white matter hyperintensities were the most commonly observed lesion in patients with ECDS/PRS and an abnormal MRI scan [8]. The exact pathophysiology of these focal white matter hyperintensities remains unclear, but it is likely they represent areas of inflammation, emphasizing their clinical relevance [12]. In line with previous studies, we have also observed a lack of MRI changes in some patients despite the presence of neurological symptoms [4]. We suggest this observation is clinically important since it emphasizes that a normal brain MRI does not exclude potentially reversible CNS involvement associated with ECDS/PRS. Better neuroimaging techniques that are capable of identifying the underlying pathological mechanisms (inflammatory and thrombotic) are needed to facilitate the diagnosis of CNS involvement in these diseases, particularly since treatment delay can be associated with high morbidity. Quantitative MRI techniques such as magnetic resonance spectroscopy (MRS), magnetisation transfer imaging (MTI), diffusion-weighted imaging (DWI), and perfusion-weighted imaging (PWI), as well as nuclear imaging techniques, such as single-photon emission computed tomography (SPECT), may have a role in detecting changes in ECDS/PRS, and could facilitate earlier diagnosis. The clinical relevance of these approaches therefore requires further validation in ECDS/PRS.

**Table 5:** Comparison of findings to previous small case series on paediatric ECDS/PRS

		Doolittle	Maloney	Chiu	Knights
**Number of patients**		23^a^	10	19^a^	14
**MRI abnormalities**	**Any**	48%^b^	100%	32%	50%
	**White matter hyperintensity**	35%	90%	16%	43%
	**Dystrophic calcification**	4%	50%	5%	36%
	**Contrast enhancement**	0%	20%	0%	36%
	**Sulcal crowding**	4%	0%	11%	21%
	**Cavernomata**	0%	0%	0%	7%
	**Porencephaly**	4%	40%	0%	7%
	**Other**	17%	0%	5%	0%
**Neurological manifestations**	**Neurological symptoms**	43%^c^	80%	42%	64%
	**Seizures**	13%	50%	16%	43%
	**Headaches**	26%	50%	16%	29%
	**Other**	9%	10%	42%	50%

^a^Only children with MRI during childhood included

^b^Bilateral findings common

^c^Only diagnosed after neurological review

Histological data were not available for our study as brain biopsy is rarely performed in these cases due to the invasiveness of the procedure. Adult studies have suggested, however, that most of the CNS lesions in ECDS/PRS are likely inflammatory, either only affecting the parenchyma, or also the CNS vasculature causing vasculitis [12]. Interestingly, on the basis of their clinical presentation with severe epilepsy and the indication of an inflammatory process on brain biopsy, some patients with PRS have been diagnosed with a variant of Rasmussen encephalitis [13].

We detected no association between the presence of an abnormal MRI brain and specific demographic, clinical, or laboratory parameters. We have, however, identified an association between white matter hypersensitivities and seizures, albeit in a smaller exploratory analysis. Future larger studies are now needed to firmly establish the relationship between clinical endophenotypes and the risk of CNS inflammation and association with detectable MRI findings.

Our study is limited by all the usual caveats around small and largely descriptive retrospective case studies, an unavoidable situation since ECDS/PRS is so rare in the young. We used standardised protocols for conventional MRI sequences (such as T1-weighted [with and without contrast agents], T2-weighted, and FLAIR images), as utilised for routine care in our institution for at least 10 years. Further characterisation of the lesions with more recently introduced techniques and gadolinium enhancement may have increased the MRI detection yield. Detailed analysis of imaging findings of patients with PRS without ECDS would be interesting and needs to be addressed in future studies. It also remains challenging to determine whether the psychiatric manifestations we describe are related to the burden of chronic illness or are a true manifestation of CNS inflammation. Lastly, the relationship between skin disease activity and the development of MRI abnormalities needs to be assessed in future studies.

## Conclusions

In summary, we observed several distinct radiographic patterns associated with ECDS/PRS but, importantly, some patients with neurological symptoms displayed no MRI brain abnormalities. Seizure disorder was strongly associated with the presence of enhanced white matter signalling ipsilateral to the cutaneous disease. Improved neuroimaging techniques that combine morphological with functional imaging may improve the detection rate of brain involvement in children with ECDS/PRS in the future.

## Supplementary Information

**Additional file 1: Table S1.** Other autoantibodies tested.

## Data Availability

The datasets used and/or analysed during the current study are available from the corresponding author on reasonable request.
