# Supplementary material for: Magnetic resonance imaging findings in children with Parry-Romberg syndrome and en coup de sabre
Source: Pediatr Rheumatol Online J. 2021 Mar 23;19:42. doi: 10.1186/s12969-021-00512-6 (PMC7986399; doi:10.1186/s12969-021-00512-6)
Supplement: Supplementary file 1 — Additional file 1: Table S1. Other autoantibodies tested. [file 12969_2021_512_MOESM1_ESM.docx]

**Supplementary Table S1. Other autoantibodies tested.**

| **Autoantibodies** | **Frequency positive** |
| --- | --- |
| **dsDNA** | 0/7 |
| **ENA** | 0/6 |
| **ANCA** | 0/6 |
| **TTG** | 0/5 |
| **TPO** | 0/5 |
| **GPC** | 0/5 |
| **Mitochondrial** | 0/5 |
| **Reticulin** | 0/5 |
| **LKM** | 0/5 |
| **Cardiolipin** | 0/3 |
| **NMDAr** | 0/1 |
| **VGKC** | 0/1 |

Double stranded DNA (dsDNA); extractable nuclear antigens (ENA); antineutrophil cytoplasmic antibodies (ANCA); tissue transglutaminase (TTG); thyroid peroxidase (TPO); gastric parietal cell (GPC); liver kidney microsomal (LKM); NMDA receptor (NMDAr); voltage-gated potassium channel (VGKC)**.**
